# A unique volatile signature distinguishes malaria infection from other conditions that cause similar symptoms

**DOI:** 10.1038/s41598-021-92962-x

**Published:** 2021-07-06

**Authors:** Hannier Pulido, Nina M. Stanczyk, Consuelo M. De Moraes, Mark C. Mescher

**Affiliations:** Department of Environmental Systems Science, ETH Zürich, 8092 Zürich, Switzerland

**Keywords:** Diseases, Infectious diseases, Biomarkers, Diagnostic markers

## Abstract

Recent findings suggest that changes in human odors caused by malaria infection have significant potential as diagnostic biomarkers. However, uncertainty remains regarding the specificity of such biomarkers, particularly in populations where many different pathological conditions may elicit similar symptoms. We explored the ability of volatile biomarkers to predict malaria infection status in Kenyan schoolchildren exhibiting a range of malaria-like symptoms. Using genetic algorithm models to explore data from skin volatile collections, we were able to identify malaria infection with 100% accuracy among children with fever and 75% accuracy among children with other symptoms. While we observed characteristic changes in volatile patterns driven by symptomatology, our models also identified malaria-specific biomarkers with robust predictive capability even in the presence of other pathogens that elicit similar symptoms.

## Introduction

The presence of disease can alter human odors, including volatile emissions from skin and breath. The potential diagnostic value of such changes in volatile chemistry has long been recognized, and volatile-based diagnostics are being actively explored for a number of diseases, including several types of cancer^1,2^. However, a major challenge for the development of volatile disease biomarkers is posed by the inherent variability of human volatile emissions, which are highly labile and can be influenced by a wide range of genetic, physiological, and environmental factors^3^. In light of this variability, there is reason to speculate that volatile biomarkers might be of particular value in diagnosing diseases caused by insect-borne pathogens, which frequently manipulate the odors of their hosts in ways that influence vector behavior and might therefore be highly conserved^4–6^. Indeed, a number of recent studies on human malaria have identified characteristic changes in the volatile emissions from the skin and breath of infected individuals^7–12^. Yet, uncertainty remains about the physiological bases of these volatile changes and the extent to which they are uniquely caused by malaria or products of pathological processes that might be shared with other disease states. Addressing this uncertainty has important implications for understanding the diagnostic value of such biomarkers, particularly for use in human populations where numerous pathological conditions are widespread and may give rise to similar symptomatology, as is often the case for malaria endemic regions.

A number of previous studies have documented malaria-induced changes in human skin^7–9^ and breath^10–12^ volatiles, as well as the volatile emissions of rodent malaria hosts^13^, while others have characterized the emissions of *Plasmodium* cells grown in vitro^14–16^. Effects of infection on host odors have also been reported to influence vector behavior, with several studies reporting increased mosquito attraction to hosts harboring transmissible stages of *Plasmodium* parasite^13,17,18^ and at least one showing enhanced attraction to host odor profiles in which individual compounds were manipulated to mimic the relative up- or downregulation caused by infection^13^.

Enhanced vector recruitment during the transmissible stage of infection is hypothesized to facilitate malaria transmission^19^, suggesting that the pathogen may benefit from actively manipulating host volatiles^20^. To the extent that *Plasmodium* parasites, or other vector-borne pathogens, alter host odors in ways that consistently influence vector attraction, they may also generate unique patterns of effects on host volatile profiles—possibly tailored to the olfactory responses of particular vector species—distinct from more general changes in volatile emissions that arise as mere byproducts of pathology. The presence of such unique signatures of infection might, in turn, facilitate the identification of pathogen-specific biomarkers capable of reliably predicting infection status even in populations where numerous pathological conditions elicit similar symptoms.

The identification of such robust biomarkers is of particular interest for malaria, which frequently occurs in populations affected by numerous other diseases and ailments that elicit similar symptoms, including fever, headaches, diarrhea, vomiting, etc., and might therefore plausibly be expected to have somewhat similar effects on underlying physiological processes that also influence volatile emissions. In the case of malaria, disease progression often entails alternating asymptomatic and symptomatic phases that may recur indefinitely if not treated. In a previous study, we reported differential up and down regulation of specific volatile organic compounds in both symptomatic and asymptomatic schoolchildren in Kenya^7^. Yet, while these characteristic changes were highly predictive of malaria infection, the specific processes by which the presence of malaria parasites alter human volatiles remain almost entirely unknown. One recent study reported that a malaria-derived isoprenoid increases the production of several monoterpenes and aldehydes in vitro^21^, but this does not account for the majority of volatile alterations observed by our and other studies^7–12^ suggesting that the underlying mechanisms are likely to be complex.

To explore the extent to which changes in the odors of malaria infected humans are associated with the presence of symptoms, we analyzed the volatile profiles of symptomatic children, using volatile data previously collected from a large field trial in Western Kenya^7^. Specifically, we compared the skin volatile profiles of symptomatic schoolchildren who tested positive for malaria to those of children presenting similar (malaria-like) symptoms, but who tested negative for malaria. We used a genetic algorithm framework to predict malaria infection and identify volatile compounds associated with disease status. Our goal was thus to discover which volatile changes might be specific to malaria, and which might be caused by infection with other illnesses or conditions.

## Results and discussion

### Volatile signatures differentiate symptomatic children with and without malaria infection

**Figure 1:**
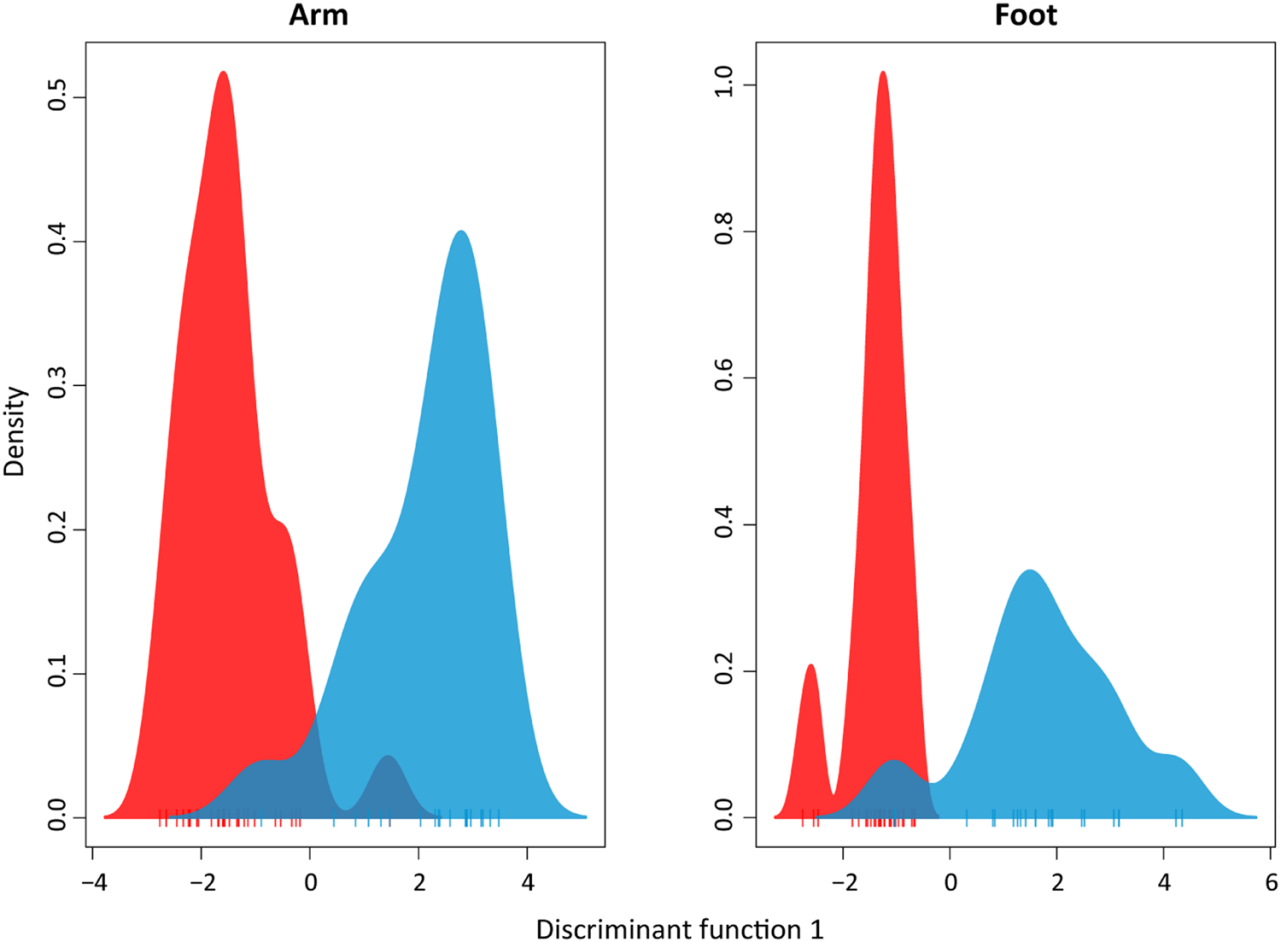
DAPC plots using the first discriminant function show separation between uninfected (blue) and malaria-infected (red) symptomatic children (exhibiting fever, diarrhea, vomiting or abdominal pain on the day of collection).

We first explored whether there was a clear signature of malaria infection among symptomatic children. In addition to examining differences in the volatile profiles of malaria-infected and uninfected children presenting any symptom (i.e., all symptomatic children), we made similar comparisons for non-exclusive subsets of children exhibiting the two most commonly observed symptoms in our dataset: fever and diarrhea. For each of these three symptom categories, discriminant analysis of principal components (DAPC) revealed clear separation in the overall volatile composition between individuals with and without malaria infection (Fig. 1, S1), with a permutational analysis of variance resulting in significant differences in both arms (pseudo*-F*_1,48_ = 2.96, *p* = 0.022) and feet (pseudo*-F*_1,49_ = 2.91, *p* = 0.003). These results indicate that the effect of malaria infection on volatile profiles is apparent even when directly compared to other conditions that produce similar symptoms.

**Figure 2:**
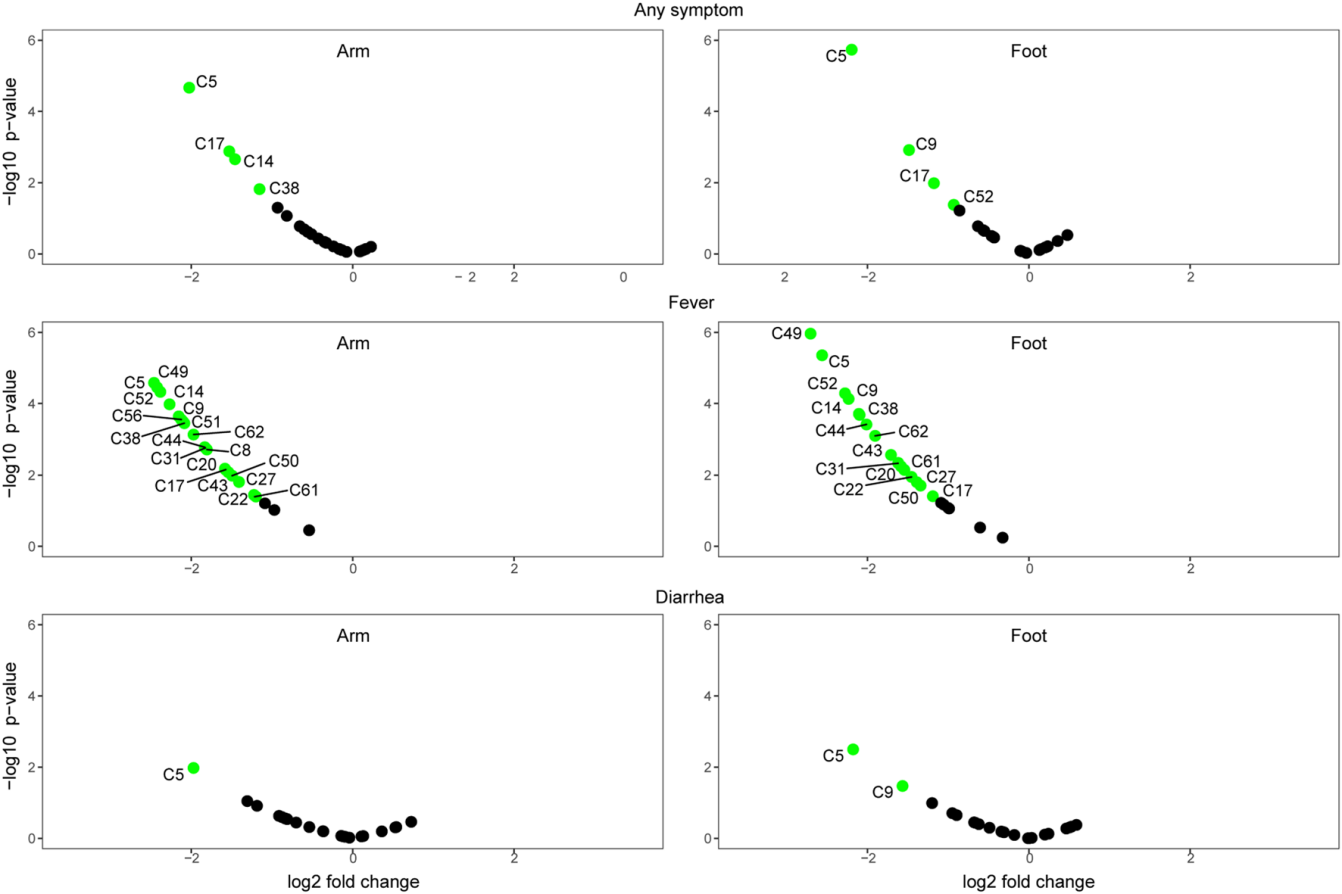
Volcano plots showing changes in individual compounds in malaria-infected symptomatic children relative to malaria-uninfected symptomatic children. Significantly up- or downregulated compounds (*p* < 0.05 and absolute fold change > 1.5) are shown in green. Compound IDs are listed in Table 2.

We next used a genetic algorithm predictive model trained to recognize malaria infection based on a subset of the data (70%) to predict malaria infection status in the remaining test set (30%). For children exhibiting any symptom, this model was able to predict malaria status with 75% accuracy (Tables 1 and 2). Given that children included in this comparison likely exhibited highly variable physiological states (i.e., different symptomatologies arising from the presence of different pathogens and various stages of disease progression) this level of predictive accuracy indicates that the volatile signature of malaria infection is relatively robust. Our model exhibited similar accuracy (75%) in predicting malaria infection among the subset of symptomatic children with diarrhea; for children with fever, however, the model predicted infections status with 100% accuracy (based using arm volatiles) (Tables 1 and 2). It also bears noting that only five compounds were required to predict malaria among children with diarrhea, compared to greater numbers of compounds required for the other symptom categories. This is consistent with the observation that few compounds show significant alteration with malaria infection status among children with diarrhea, compared to more extensive differences observed for children with fever (Fig. 2).

**Table 1:** Performance of trained models and 95% confidence intervals when used to predict symptomatic malaria infected (SM) vs. symptomatic uninfected (SU) children in the test set.

	Any sympt	Any sympt	Fever	Fever	Diarrhea	Diarrhea
	Arm	Foot	Arm	Foot	Arm	Foot
Accuracy %	75 (43, 94)	66.7 (35, 90)	100 (54, 100)	83.3 (36, 99)	75 (19, 99)	75 (19, 99)
Sensitivity %	85.7 (42, 99)	57.1 (18, 90)	100 (40, 100)	100 (40, 100)	100 (16, 100)	50 (1.2, 99)
Specificity %	60 (17, 95)	80 (28, 99)	100 (16, 100)	50 (1, 99)	50 (1.3, 99)	100 (16, 100)
Top Predictors	C5	C8	C8	C5	C8	C9
	C9	C31	C9	C8	C9	C17
	C12	C44	C17	C12	C17	C51
	C15	C49	C27	C15	C52	C52
	C17	C50	C50	C20		
	C20	C51	C51	C27		
	C22	C52	C52	C31		
	C38	C55	C56	C38		
	C44	C56		C44		
	C49	C62		C49		
	C50			C50		
	C52			C51		
	C62			C52		
				C55		
				C62		

SM with any symptom n = 29, fever n = 18, diarrhea n = 11. SU with any symptom n = 22, fever n = 11, diarrhea n = 9.

**Table 2:** Compound IDs and selected key compounds.

	Compound ID
C5	Toluene
**C8**	**Octane**
**C9**	**Hexanal**
C12	2,4-dimethylheptane
C14	Ethyl cyclohexane*
C15	2,4-dimethylhept-1-ene
**C17**	**4-hydroxy-4-methylpentan-2-one**
C20	Ethylbenzene
C22	*m*-xylene or *p*-xylene
**C27**	***o*-xylene**
C31	Unidentified
C38	Propylcyclohexane
C43	1-ethyl-3-methylbenzene
C44	Benzaldehyde
C49	Unidentified
C50	1,2,4-trimethylbenzene
**C51**	**Decane**
**C52**	**Octanal**
C55	*S*(-)-limonene
**C56**	**2-ethylhexan-1-ol**
C61	Nonanal*
C62	Dodecane

Compounds in bold are important predictors of malaria status for children with fever/diarrhea. Asterisks (*) indicate compounds that are key predictors of symptom presence/absence in children without malaria.

The higher level of predictive accuracy for children with fever may reflect distinct features of the pathology occurring in malaria-infected children. As *Plasmodium* completes its lifecycle within the host, fever is typically associated with the rupture of mature schizont cells and the release of merozoites that then reinvade red blood cells^22^. The resulting intermittent cyclic fever may cause unique physiological effects compared to other ailments, and may also play a role in the downregulation of volatiles, including the compounds that drive the predictive accuracy of our model (Fig. 2). Indeed, it is notable that malaria symptomatic children show decreased volatile production compared to both malaria-free febrile and asymptomatic children (Fig. 2, S3), as fever might otherwise be presumed to cause a general upregulation of volatile emissions due to increased body temperature and sweating causing increased evaporation of compounds from the skin.

Of the eight volatile compounds selected by our model to predict malaria status from the arms of children with fever (Tables 1 and 2), hexanal (C9), decane (C51) and octanal (C52), have been identified as predictors of malaria infection in several previous studies^7,8,15,16^. In the current study, these compounds were also important predictors of infection status among children exhibiting any symptom, as well as among the subset of children with diarrhea. Hexanal and octanal are also known to serve as host-location cues for mosquitoes, eliciting attraction or repellence depending on their concentration^23,24^. It is thus intriguing that the emission of these compounds appears to be specifically influenced by the presence of malaria parasites. As we have previously speculated^3,7^, malaria-induced changes in volatile cues that enhance transmission probability via effects on mosquito attraction^19^ might generate robust biomarkers of infection status.

### The presence of symptoms alters volatile profiles in malaria-free children

To explore how the presence of symptoms itself influences volatile emissions, we next used predictive models, similar to those described above, to examine differences between the volatile profiles of symptomatic and asymptomatic children who tested negative for malaria infection. Here our models were able to predict the presence of symptoms with 60–80% accuracy across the three symptom categories described above (fever, diarrhea, any symptom) and using either foot or arm volatiles (Table 3). Several compounds that were important predictors for the presence of symptoms in these models were also important predictors of malaria infection status among symptomatic children in our previous analyses, including C8 (octane), C9 (hexanal) and C17 (4-hydroxy-4-methylpentan-2-one), (Table 1).

**Table 3:** Performance of trained models and 95% confidence intervals when used to predict malaria-uninfected symptomatic (SU) vs. malaria-uninfected asymptomatic (ASU) children in the test set.

	Any sympt	Any sympt	Fever	Fever	Diarrhea	Diarrhea
	Arm	Foot	Arm	Foot	Arm	Foot
Accuracy %	62.5 (24, 91)	77.8 (40, 97)	80 (28, 99)	66.7 (22, 95)	60 (15, 94)	83.3 (36, 99)
Sensitivity %	80 (28, 99)	100 (47, 100)	50 (1.2, 98)	0 (0, 84)	50 (1.2, 98)	50 (1.2, 98)
Specificity %	33.3 (0.8, 90)	50 (6.7, 93)	100 (29, 100)	100 (39, 100)	66.7 (9, 99)	100 (39, 100)
Top Predictors	C8	C5	C14	C5	C9	C5
	C12	C8	C17	C8	C12	C15
	C15	C9	C22	C14	C14	C17
	C17	C12	C49	C20	C15	C20
	C20	C14		C22	C17	C22
	C22	C17		C27	C44	C27
	C38	C20		C38	C49	C43
	C51	C27		C51	C50	C49
	C52	C43		C61	C52	C50
	C61	C50		C62	C56	
	C62	C56			C62	
		C61				
		C62				

SU with any symptom n = 22, fever n = 11, diarrhea n = 9. ASU n = 16.

**Figure 3:**
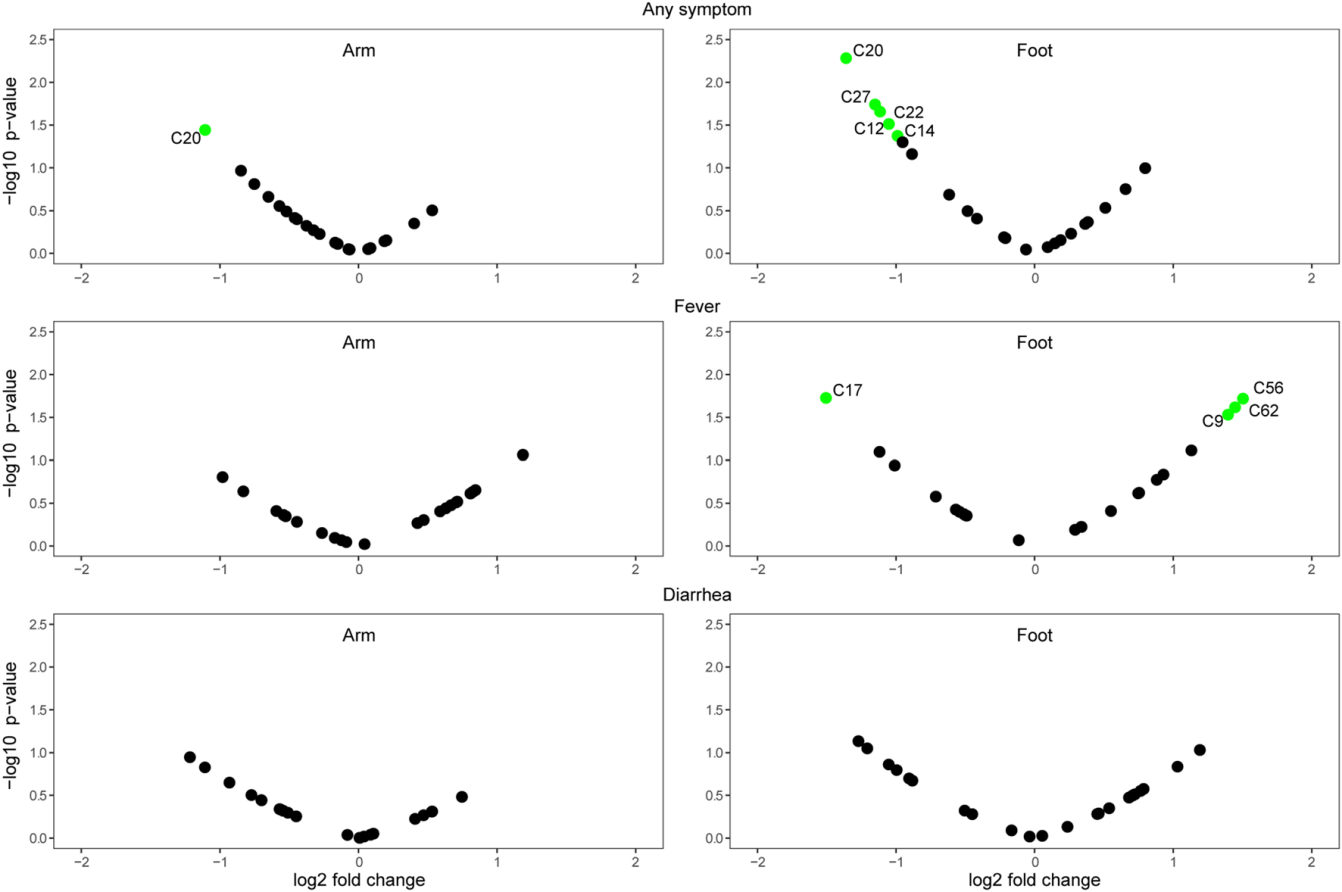
Volcano plots showing changes in individual compounds in malaria-uninfected symptomatic children relative to malaria-uninfected asymptomatic children. Significantly up- or downregulated compounds (*p* < 0.05 and absolute fold change > 1.5) are shown in green. Compound IDs are listed in Table 2.

Comparing the volatile emissions of malaria-free children exhibiting any symptom to those without symptoms revealed significant downregulation of several compounds (Fig. 3), with ethylbenzene (C20) showing the greatest downregulation in the presence of symptoms for both arm and foot volatiles. When focusing on individual symptoms, malaria-free children with diarrhea showed no significant changes in compound levels compared to those without symptoms, whereas febrile children without malaria showed an upregulation of several foot volatile compounds (Fig. 3), in contrast to the downregulation of compounds seen in febrile children with malaria compared to malaria-free children without symptoms (Fig. S3). The only compound downregulated in febrile children without malaria was C17 (4-hydroxy-4-methylpentan-2-one) (Fig. 3), which was further downregulated in malaria-infected children compared to febrile children without malaria (Fig. 2). Among upregulated compounds in malaria-free febrile children, C9 (hexanal) was previously reported to be downregulated in malaria-infected vs uninfected children^7^. The overlap in compounds identified as important predictors of malaria infection and of symptoms in malaria-free children (but with different patterns of up and down regulation) is intriguing and may reflect the influences of different pathological conditions on the same underlying physiological processes.

### Overlap in volatile biomarkers for malaria and other diseases

As noted above, the use of volatile diagnostics is under investigation or optimization for a number of diseases, including various types of cancer and pneumonia, and many studies have examined the volatiles in breath that change dependent on disease status^1,2,25^. In the case of lung cancer, a large-scale trial for diagnostic detection using VOCs is currently underway^26^. For many other diseases, however, significant challenges remain to be overcome prior to the implementation of large-scale trials, including the identification of a robust and reproducible set of candidate biomarkers^3^, which can be complicated by the absence of standardized methods for volatile collection and analysis^27^. In the case of malaria, several previous studies have reported successful prediction of infection status based on analysis of VOCs; however, there is considerable variation in the predictive compounds identified, likely owing to divergent methodologies across studies. Two compounds, hexanal and nonanal, have consistently been found informative with respect to with malaria status, each reported in three separate studies from skin and breath volatiles as well as in the prediction here of malaria status in symptomatic children (Table 4). Among compounds identified as predictors of malaria infection in the current study, hexanal and nonanal, along. with decane and octanal, have previously been implicated as disease predictors in studies on other diseases in addition to malaria (Table 4). Hexanal, nonanal, octanal and have been identified in the breath of lung and (along with decane) breast cancer patients, while nonanal has also been identified as a predictor of colorectal cancer and pneumonia^2,25^. A further comparison of the key predictive compounds from this study and those identified for other diseases reveals several compounds that are specific to the differentiation of infection status for malaria, including octane, 4-hydroxy-4-methylpentan-2-one, o-xylene and 2-ethylhexan-1-ol. This specificity may be related to the efficacy of these compounds in distinguishing malaria infections form other conditions that give rise to similar symptoms; however, this remains speculative given that many of the other diseases for which volatile diagnostics have been studied do not display similar symptoms. Finally, the recurrence of certain compounds such as hexanal and nonanal as predictors of multiple diseases may, again, reflect the influences of different pathological conditions on the same underlying processes, but with disease-specific patterns of effects on volatile emissions that can give rise to distinct signatures.

**Table 4:** Volatiles altered by malaria in previous studies, and other diseases (bold).

Skin				Breath			In vitro			Mice
Current	de Boer 2017	De Moraes 2018	Robinson 2018	Berna 2015	Shaber 2018	Berna 2018	Kelly 2015	Correa 2017	Capuano 2019	De Moraes 2014
Octane	(R)- or (S)-2-methylbutanal	**Toluene**	**Heptanal**	CO2	Methyl undecane	α-terpinene	n-butane	**Hexanal**	**Hexanal**	Tridecane
**Hexanal**	**(R)- or (S)-3-methylbutanal**	**Hexanal**	**Octanal**	**Isoprene**	Dimethyl decane	m-cymene	n-hexane		Styrene	N,N-dibutylformamide
4-hydroxy-4-methylpentan-2-one	**(R)- or (S)-3-hydroxy-2-butanone**	Ethylcyclohexane	**Nonanal**	Acetone	Trimethyl hexane	**Limonene**	**Toluene**		**Ethylbenzene**	2-pyrrolidone
*o*-xylene	6-methyl-5-hepten-2-one	4-hydroxy-4-methylpentan-2-one	(E)-2-octenal	Benzene	**Nonanal**	Terpinolene	2,3-dimethyl heptane			3-methyl-2-buten-1-ol
**Decane**	1-dodecene	Ethylbenzene	(E)-2-decenal	**Cyclohexane**	I**soprene**	Allyl methyl sulfide	1,4-dimethyl-trans-cyclooctane			3-methyl butanoic acid
**Octanal**	Dodecanal	Propylcyclohexane	2-octanone	Allyl methyl sulfide	Tridecane	1-methylthio-propane				2-hexanone
2-ethylhexan-1-ol	Methyl dodecanoate	2-ethylhexan-1-ol		1-methylthio-propane	α-pinene	(Z)-1-methylthio-1-propene				**Benzaldehyde**
**Nonanal**		**Nonanal**		(Z)-1-methylthio-1-propene	**3-carene**	(E)-1-methylthio-1-propene				
Ethylcyclohexane		**Dodecane**		(E)-1-methylthio-1-propene						
		**Benzaldehyde**								
		**Decane**								

Lung cancer: hexanal, octanal, nonanal, 3-hydroxy-2-butanone, 3-methyl-butanal (in vitro)^28–30^.

Breast cancer: hexanal, heptanal, octanal, nonanal, decane, limonene, cyclohexane^31,32^.

Colorectal cancer: nonanal^33^.

Prostate: Toluene^34^.

Head and neck cancer: isoprene, limonene^35,36^.

Pneumonia: nonanal, ethylbenzene, benzaldehyde, 3-methyl-butanal, cyclohexane, dodecane, 3-carene^37–40^.

## Conclusion

Volatile biomarkers hold significant promise for the development of non-invasive techniques for disease diagnosis^1^. However, extensive variation in human volatile emissions, including that due to the presence of many different diseases and ailments within human populations, poses a significant challenge. While the robustness of malaria biomarkers across varying genetic and environmental backgrounds still needs to be assessed in large-scale trials before they could enter clinical use, the current findings indicate that volatile biomarkers can identify malaria infection even in the face of variation elicited by the presence of other symptomatic conditions. Our predictive models identified malaria cases with 100% accuracy for children with fever and with 75% accuracy for other symptoms. We also identified specific compounds that are important predictors of malaria infection among symptomatic children, as well as compounds that are more generally indicative of the presence of symptoms. These results suggest that, while some changes in human volatile profiles are broadly associated with the presence of symptomatic disease, malaria elicits specific changes in key compounds that provide a unique signature of infection.

## Methods

### Ethics approval and participant selection

This study was approved by The Pennsylvania State University (IRB #41,529), ETH Zürich (EK2015-*N*-59), and the Kenya Medical Research Institute (SERU 391) and all experiments were performed in accordance with relevant guidelines and regulations. Before sample collection, the study and consent form were explained to parents/guardians and their written informed consent was obtained.

Participant exclusion criteria included (1) receipt of antimalarial medication during the previous 2 wk; (2) chronic disease, such as HIV; (3) not signing (or having a parent sign) the consent form; and (4) refusal of malaria treatment.

### Symptom categories

Our analyses were performed on data derived from skin volatile samples (1 h collections from arms and feet) collected from students at 41 primary schools near Mbita Point, Kenya between 2013 and 2016^7^. Symptoms were self-reported in an initial interview using a standardized questionnaire; symptoms indicative of malaria included fever, abdominal pain, rash, diarrhea, vomiting and body aches. For our analysis, we sorted children into the categories (1) any symptom (2) fever, (3) diarrhea and (4) asymptomatic. The any symptom category comprised children with fever, diarrhea, abdominal pain or vomiting; the fever and diarrhea categories were non-exclusive subsets of the any symptom category. Abdominal pain and vomiting were not analyzed independently due to low numbers of children with these symptoms. Malaria infection status was initially assessed via rapid diagnostic testing (SD Bioline), then confirmed by light microscopy and PCR. For the current study, children in the malaria infected category tested positive by both microscopy and PCR.

The dataset used for this study comprised volatile profiles for 114 children. Once categorized into symptoms for the below analyses, numbers of children in each category were as follows: malaria infected children with any symptom = 29, fever = 18, diarrhea = 11. Malaria-free children with any symptom = 22, fever = 11, diarrhea = 9, asymptomatic = 16.

### Volatile data

Arm and foot volatiles were collected simultaneously for 1 h by enclosing the arm (from wrist to above the elbow) or foot (to below the knee) in a teflon bag, pushing filtered air through an entry port (arm: 1.1 L/min, foot: 1.8 L/min) and pulling it through an exit port (arm: 0.8 L/min, foot: 1.1 L/min) where it was collected on an adsorbent HaySepQ filter. Compounds trapped on filters were then eluted with 150ul dichloromethane and analyzed by GC–MS (Full methods: De Moraes 2018^7^).

### Statistical analyses

Discriminant Analysis of Principal Components (DAPC)^41,42^ was used to compare the separation of healthy from malaria-infected individuals. These group differences were tested using a permutational analysis of variance (PERMANOVA) with the Euclidean similarity on the scaled data^43^.

We used a Genetic Algorithm (GA) to predict disease condition and to select one or more sets of compounds for each of the symptom subsets to be assessed as a potential biomarker for malaria. Each subset was randomly split into 70% for model training and 30% for validation. Using the training set, each model was trained five times using a repeated tenfold cross-validation. Genetic Algorithms are feature selection procedures that are conceptually based on the principle of evolution by natural selection^44^. They have been used as a promising multivariate approach in the analysis of metabolomics datasets^45,46^. The algorithm works by evolving initial sets of variables (chromosomes) from a random population via cycles of replication, recombination and mutation of the fittest chromosomes. The iterations were repeated for 100 generations, with a population size of 50 candidate solutions for each model and crossover and mutation probabilities of 0.8 and 0.1, respectively. The performance of the fittest GA model was used to predict the samples in the validation set. To complement the predictive models, a differential analysis between infection statuses was performed using the limma R package^47^. The GA models were formulated with the caret R package^48^.

## Supplementary Information

Supplementary information.

## Data Availability

All relevant data reported in this paper have been deposited in ETH Zurich’s Research Collection, http://hdl.handle.net/20.500.11850/458605 (https://doi.org/10.3929/ethz-b-000458605).
