# Supplementary material for: A unique volatile signature distinguishes malaria infection from other conditions that cause similar symptoms
Source: Sci Rep. 2021 Jul 6;11:13928. doi: 10.1038/s41598-021-92962-x (PMC8260776; doi:10.1038/s41598-021-92962-x)
Supplement: Supplementary file 1 — Supplementary information. [file 41598_2021_92962_MOESM1_ESM.docx]

**Supplementary Material**

**Title:** **A unique volatile signature distinguishes malaria infection from other conditions that cause similar symptoms**

**Authors:** Hannier Pulido^1^, Nina M. Stanczyk^1^, Consuelo M. De Moraes^1^, Mark C. Mescher^1†^

**Affiliations:**

1. Department of Environmental Systems Science, ETH Zürich, Zürich, 8092, Switzerland

^†^To whom correspondence should be addressed:

Mark C. Mescher, Department of Environmental Systems Science, Institute of Integrative Biology, LFO G19, ETH Zürich, 8092 Zürich, Switzerland

Email address: [mescher@usys.ethz.ch](mailto:mescher@usys.ethz.ch), Telephone number: +41 44 632 39 30, Fax: +41 44 632 11 71

Keywords: malaria, symptomatic infection, symptomology, diagnostics, disease biomarkers, volatiles, Genetic algorithm


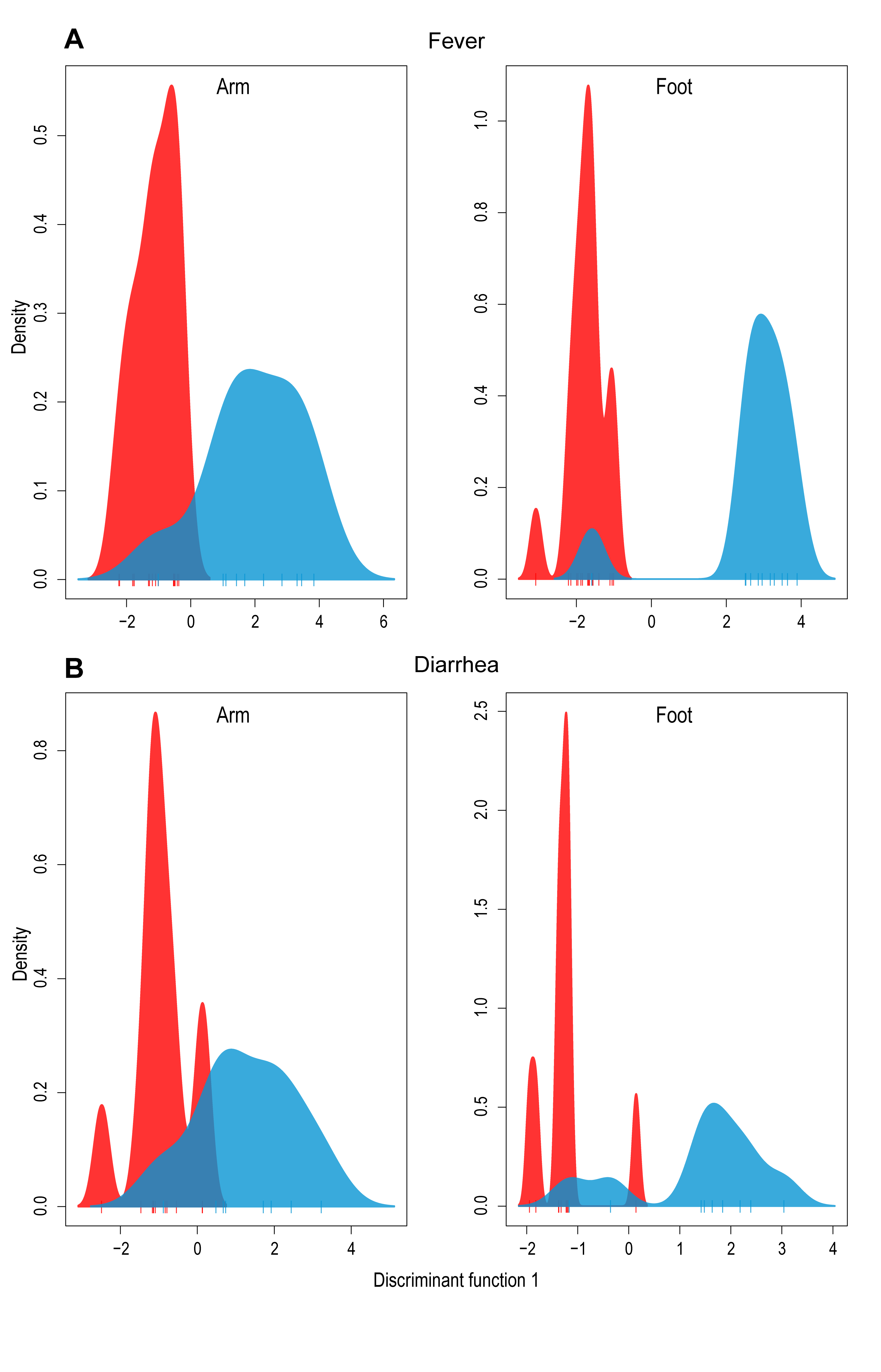


**S1 Fig.** DAPC plots using the first discriminant function show separation between **symptomatic malaria-infected (red) and malaria-free (blue) children along the first discriminant function**. DAPC analysis of volatiles from the arm and feet of children exhibiting either Fever (A) or diarrhea (B) on the day of collection.


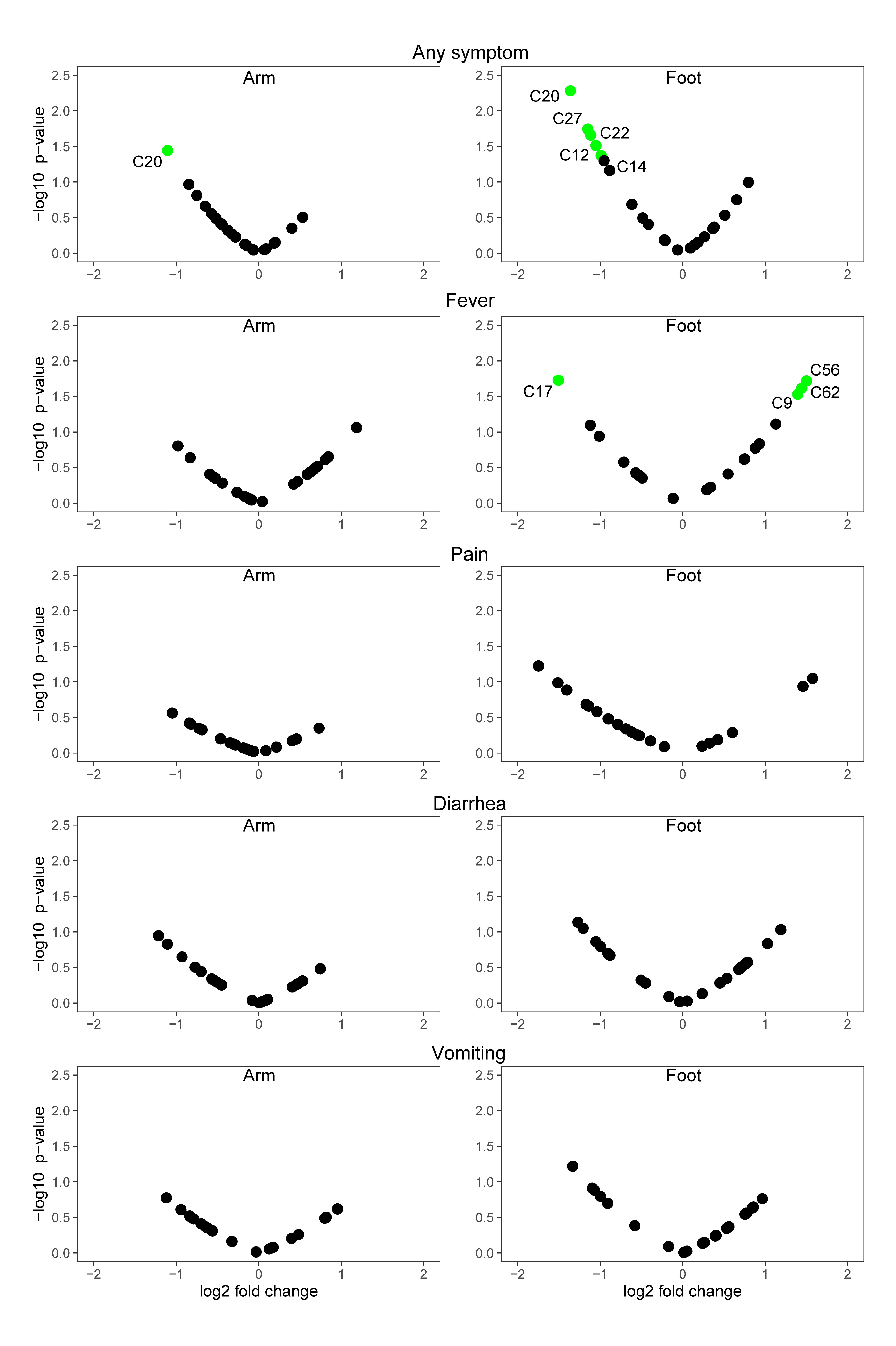


Fig S2. Volcano plots showing changes in individual compounds in malaria-free symptomatic children relative to asymptomatic children. Significantly up- or downregulated compounds (p<0.05 and absolute fold change >1.5) are shown in green.


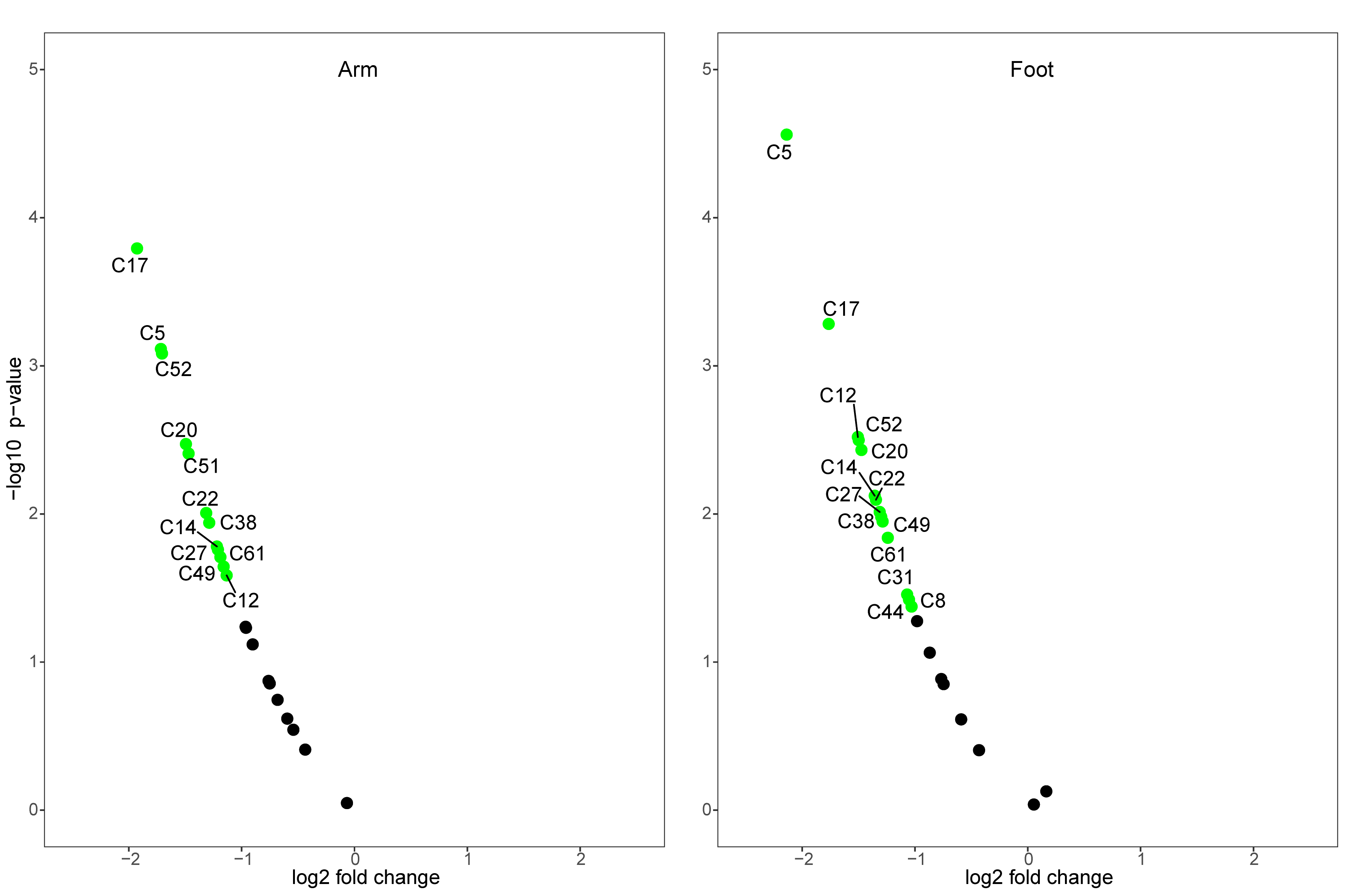


Fig S3. Volcano plot showing changes in individual compounds in febrile malaria-infected relative to malaria-free asymptomatic children. Significantly up- or downregulated compounds (p<0.05 and absolute fold change >1.5) are shown in green.
